# Ambra1 Alleviates Hypoxia/Reoxygenation Injury in H9C2 Cells by Regulating Autophagy and Reactive Oxygen Species

**DOI:** 10.1155/2020/3062689

**Published:** 2020-10-06

**Authors:** Lin Zhao, Liting Cheng, Yongquan Wu

**Affiliations:** Department of Cardiology, Beijing Anzhen Hospital, Capital Medical University, Beijing, China

## Abstract

Reperfusion therapy is the most important method for treating acute myocardial infarction. However, myocardial ischemia reperfusion injury (MIRI) can offset the benefit of reperfusion therapy and worsen the outcome. In both ischemia and reperfusion, autophagy remains problematic. Activating molecule in Beclin1-regulated autophagy (Ambra1) is an important protein in autophagy regulation, and its function in MIRI remains unclear. Thus, we used H9C2 cells to investigate the function of Ambra1 in MIRI and the underlying mechanisms involved. Hypoxia and reoxygenation of H9C2 cells were used to mimic MIRI in vitro. During hypoxia, autophagy flux was blocked, then recovered in reoxygenation. Ambra1 overexpression increased autophagy in the H9C2 cells, as the LC3B II/I ratio increased, and alleviated cellular necrosis and apoptosis during hypoxia and reoxygenation. This effect was counteracted by an autophagy inhibitor. Knocking down Ambra1 can block autophagy which P62 sediment/supernatant ratio increased while the ratio of LC3B II/I decreased, and worsen outcomes. Ambra1 enhances autophagy in H9C2 cells by improving the stability and activity of the ULK1 complex. Reactive oxygen species (ROS) are an important cause of MIRI. ROS were reduced when Ambra1 was overexpressed and increased when Ambra1 was knocked down, indicating that Ambra1 can protect against hypoxia and reoxygenation injury in H9C2 cells by promoting autophagy and reducing ROS.

## 1. Introduction

Acute myocardial infarction is the leading cause of death worldwide [1]. As morbidity from acute myocardial infarction gradually increases, early reperfusion therapy has become the most important method for saving these patients' lives [2]. Reperfusion therapy refers to the recovery of blood perfusion in the ischemic area through drug thrombolysis, stent implantation, or thrombectomy [3, 4]. However, myocardial ischemia reperfusion injury (MIRI) counteracts the benefits of reperfusion therapy and even worsens the outcomes from acute myocardial infarction [5, 6]. During the process of reperfusion therapy on acute ischemic myocardium, some patients undergoing reperfusion therapy may increase the possibility of myocardial cell death and the area of myocardial infarction. Reperfusion therapy is an independent factor that caused myocardial cell death [7–9]. Resolving or relieving both ischemia and reperfusion injury remains a challenge. Ischemia leads to many cellular changes, including alterations in membrane potential, ion distribution (increasing influx of Ca^2+^ and Na^+^), cell swelling and cytoskeletal disorganization, and cellular acidosis [10, 11]. Autophagy is necessary for maintaining the cellular environmental homeostasis during ischemia injury. The underlying mechanisms involved in MIRI remain unclear, but causes of MIRI can include Na^+^ and Ca^2+^ accumulation, decreases in pH, mitochondrial permeability transition pore (mPTP) dissipation, increased reactive oxygen species (ROS) and free radical formation, and nitric oxide (NO) metabolic disorder, and autophagy is also very important in MIRI [12–15].

Autophagy is the cellular process of digesting damaged proteins and organelles and maintaining cellular environmental homeostasis and is very important under both physiological and pathological conditions [16]. Autophagy includes three forms: macroautophagy, microautophagy, and chaperone-mediated autophagy (CMA) [17, 18]. At present, macroautophagy (hereinafter referred to as autophagy) is the most widely studied in the heart. Under physiological or pathological conditions, autophagosomes are formed, and related substances (such as cell proteins, debris, or organelles) are wrapped and transported to lysosomes, autophagosomes and lysosomes form autophagolysosomes, which are then degraded [19]. Activating molecule in Beclin1-regulated autophagy (Ambra1) is a highly conserved adapter protein in vertebrates and has been showed that it plays many important roles in many aspects in cell proliferation, nervous system development during embryogenesis and autophagy modulation under physiological and pathological conditions [20, 21]. In autophagy, Ambra1 enhances mTORC1-dependent autophagosome core complex formation [22, 23]. Ambra1 is also important in selective autophagy and regulates both PARKIN-dependent and -independent mitophagy [24]. Furthermore, Ambra1 was recently reported to reduce oxidative stress through mitophagy [25].

Autophagy is very important during both ischemia and reperfusion; however, the effect of autophagy in MIRI remains controversial. Some studies have shown that enhancing autophagy can protect the heart from ischemia and reperfusion [26–28], while others have yielded contradictory outcomes [29, 30]. Therefore, we investigated the function of Ambra1 in MIRI using a model of H9C2 cells hypoxia and reoxygenation to mimic it. Ambra1 has been reported to regulate ROS, and ROS is an important cause of MIRI [12, 25, 31]; thus, regulating ROS may also change the outcomes of myocardial ischemia and reperfusion. Therefore, we investigated the role that Ambra1 plays in myocardial ischemia and reperfusion and its mechanisms.

## 2. Materials and Methods

### 2.1. Cell Cultures

H9C2 cells were obtained from the American Type Culture Collection (ATCC; USA) and cultured in Dulbecco's modified Eagle's medium (DMEM; Sigma-Aldrich, USA) supplemented with 10% fetal bovine serum (FBS; Gibco Life Technologies, USA), 100 U/ml penicillin, and 100 *μ*g/ml streptomycin (HyClone, USA) at 37°C in a 5% CO_2_ incubator. Cells were passaged when they reached 90% confluence.

### 2.2. Adenovirus Transfection

H9C2 cells were cultured in complete medium, and control adenovirus, Ambra1-overexpression adenovirus, and shAmbra1 adenovirus with GFP flag (Hanbio, China) were added, respectively, when the cell density reached 50–70%. After 8 h, the medium was changed to fresh complete medium. At 72 h after adenovirus transfection, GFP and Western blot were analyzed to confirm that the adenovirus was transfected successfully; then, downstream experiments were performed.

### 2.3. Hypoxia and Reoxygenation Treatment

To mimic ischemia and reperfusion, the primary culture medium was discarded and washed with phosphate-buffered saline (PBS; Solarbio, China). The H9C2 cells culture medium was replaced with DMEM without glucose or FBS, then subsequently placed in a hypoxia incubator with 1% O_2_ for 6 h. Next, the medium was replaced with normal complete medium and cultured in a standard cell culture incubator for 4 h (5% CO_2_, 37°C).

### 2.4. Western Blot

H9C2 cells were lysed with RIPA buffer mixed with proteinase inhibitor cocktail (Abcam, Britain) and PMSF (Sigma, USA). The cell extract was centrifuged at 12000 × g for 30 min at 4°C. Supernatant concentrations were measured using a BCA Protein Quantitation Kit (Thermo Fisher Scientific, USA). The concentration of the protein precipitate was proportional to the supernatant. After denaturation, both the supernatants and precipitates were separated via SDS-PAGE, then transferred to a polyvinylidene fluoride (PVDF) membrane (Millipore, USA). Membranes incubated in the corresponding antibodies and stored at 4°C overnight. Anti-LC3B antibody and anti-P62 antibody were purchased from Abcam (Britain). Anti-Ambra1 antibody was purchased from Cell Signaling Technology (USA). Anti-*β*-actin antibody was purchased from Solarbio (China). After washing with TBST (Solarbio, China), the secondary antibody (Abcam, Britain) was incubated. Finally, the membranes were visualized using the Gel Doc XR+ System (Bio-Rad, USA).

### 2.5. Confocal Microscopy

H9C2 cells cultured on slides were washed with PBS, then fixed with 4% paraformaldehyde (Solarbio, China) for 1 hour. After fixation, PBS was used to wash the redundant paraformaldehyde three times for 10 min each time. Subsequently, cells were permeabilized with 0.5% Triton X-100 (Sigma, USA) for 10 min, then washed again with PBS. After blocking in 1% BSA (Sigma, USA), cells were incubated with primary antibodies overnight at 4°C, then visualized using secondary antibodies under a confocal microscope (Olympus FLUOVIEW FV1000, Japan).

### 2.6. Trypan Blue Assay

H9C2 cells digested in prewarmed trypsin solution (Solarbio, China) were mixed in the trypan blue solution (Solarbio, China). After mixing for three minutes, 20 *μ*l of cell suspension was transferred to a cell-counting chamber, then inserted into a cell-counting machine (TC20TM Automated Cell Counter, USA) to analyze cell viability.

### 2.7. Cell Apoptosis Detection

Culture media was removed from the dishes, and H9C2 cells were washed once with PBS. Prewarmed trypsin solution without EDTA (Solarbio, China) was added to digest the cells from the dishes. Culture media with 10% FBS was added to neutralize the extra trypsin solution. Cell suspensions were transferred to tubes and centrifuged at 1000 rpm for 3 minutes. The supernatant was then discarded, and the cells were resuspended with 1× binding buffer. Next, 5 *μ*l Annexin-APC (Abcam, Britain) and PI (Abcam, Britain) were added to 100 *μ*l cell suspension, then incubated in the dark for 15 minutes at room temperature. After adding 200 *μ*l of 1× binding buffer, the cell suspension was analyzed via flow cytometry (BD FACSCalibur, USA).

### 2.8. Superoxide Anion Detection

Dihydroethidium (DHE; Beyotime, China) was dissolved in DMSO, then diluted with complete medium into working solution. H9C2 cells were made into a cell suspension with 200 *μ*l working solution as above, then incubated in the dark for 30 min at 37°C. After incubation, the cells were washed with complete medium, then analyzed via flow cytometry.

### 2.9. Statistical Analysis

The SPSS 21 software was used to perform the statistical analyses. One-way analysis of variance was used to analyze the differences between multiple groups, and Student's *t*-test was used to evaluate the differences between two groups. All data are presented as the means ± S.D. of at least three independent experiments. *P* < 0.05 was considered statistically significant.

## 3. Results

### 3.1. Ambra1 Overexpression Promoted Autophagy in H9C2 Cells, While Ambra1 Deficiency Blocked Autophagy Flux

**Figure 1:**
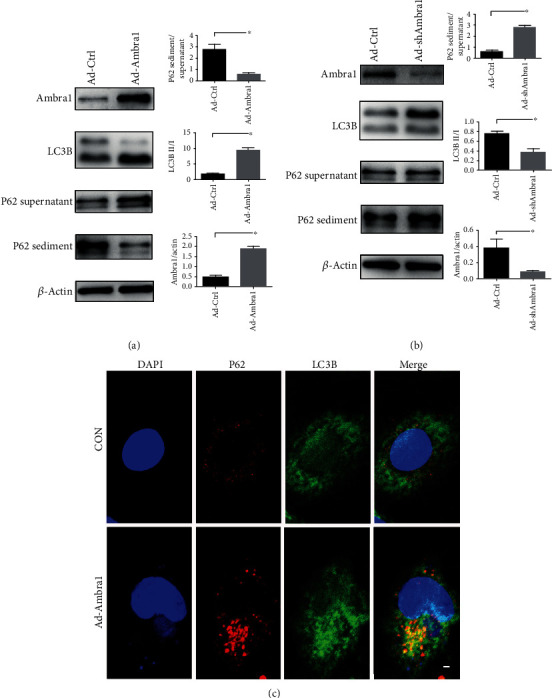
Ambra1 overexpression promotes autophagy in H9C2 cells under basal condition while knockdown of Ambra1 blocks the autophagy flux. (a, b) Representative Western blot and quantitative evaluation of the expression of P62 sediment/supernatant, LC3B II/I, and Ambra1/actin. (c) Representative confocal image of P62 and LC3B in H9C2 cells. Scale bar: 5 *μ*m; ^∗^*P* < 0.05.

To determine the function of Ambra1 in autophagy in H9C2 cells, we constructed control adenovirus (Ad-Ctrl), Ambra1-overexpression adenovirus (Ad-Ambra1), and shAmbra1 adenovirus (Ad-shAmbra1), then transinfected these adenoviruses into H9C2 cells. At 72 h after transfection, Western blot was used to examine the Ambra1 expression and autophagy flux. After Ad-Ambra1 was transinfected, Ambra1 was successfully overexpressed, and LC3B detection showed that the LC3B type I conversion to type II was increased, and the P62 supernatant was increased, while the P62 sediment was decreased (Figure 1(a)). This change indicated that in the H9C2 cells under basal conditions, Ambra1 overexpression contributed to autophagy. Conversely, when Ambra1 was knocked down in H9C2 cells, the ratio of LC3B II/I decreased, and the P62 sediment/supernatant increased (Figure 1(b)), indicating autophagy flux block [32]. This outcome was verified via confocal microscopy after Ambra1 overexpression compared with normal H9C2 cells. More LC3B (green) and P62 (red) were expressed and gathered around the nucleus (blue), indicating autophagosome formation (Figure 1(c)). Thus, Ambra1 expression contributed to autophagy in H9C2 cells.

### 3.2. Autophagy Was Blocked during Hypoxia and Restored during Reoxygenation

**Figure 2:**
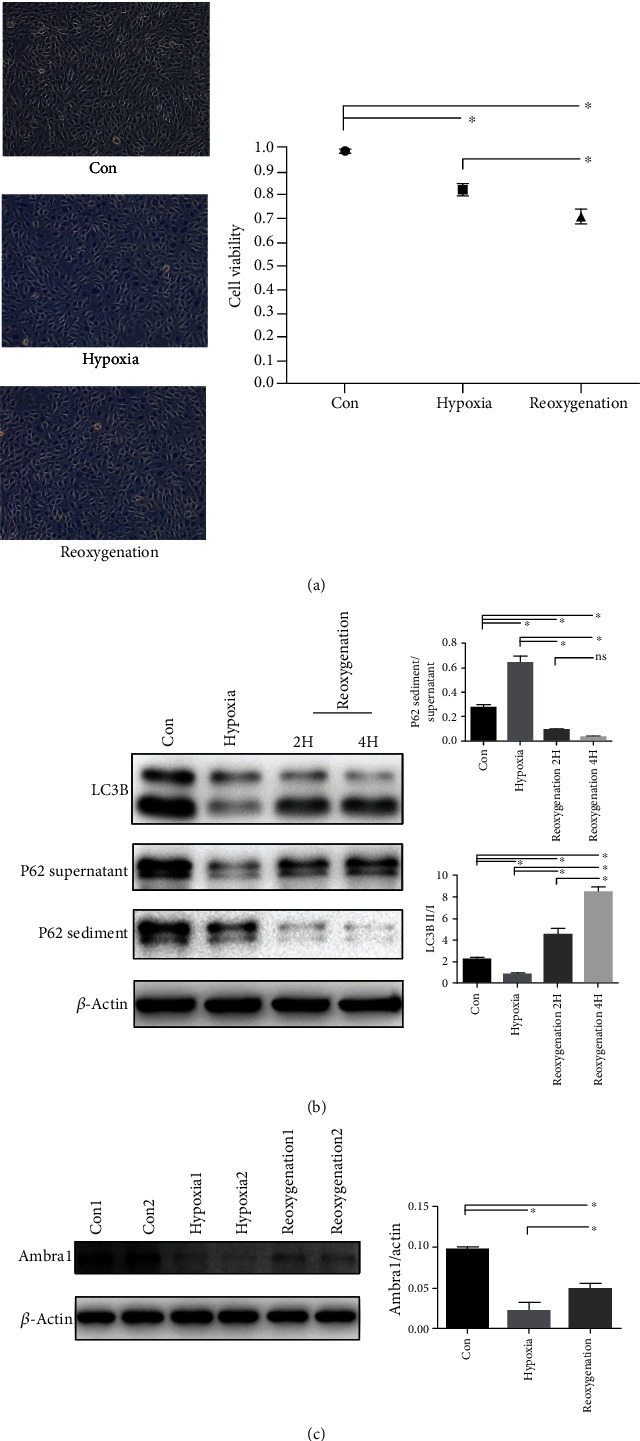
Cell viability decreases in H9C2 cell hypoxia and reoxygenation model, and autophagy flux is blocked during hypoxia then restored after reoxygenation. (a) Representative image of cell viability detected by trypan blue staining and statistics analysis. (b) Representative Western blot and quantitative evaluation of the expression of P62 sediment/supernatant and LC3B II/I. (c) Representative Western blot and quantitative evaluation of the expression of Ambra1 change. ^∗^*P* < 0.05; ns: no statistic difference.

To determine the optimum time for hypoxia and reoxygenation in H9C2 cells, several conditions were set, which revealed 6 hours of hypoxia, and then 4 hours of reoxygenation was the best. After 6 h of hypoxia and 4 h of reoxygenation, trypan blue staining was used to detect the cell viability. Cell viability was decreased during both hypoxia and reoxygenation (Figure 2(a)). Western blot was used to detect autophagy flux by testing for LC3B and P62 in the supernatant and sediment. During hypoxia, the P62 sediment/supernatant ratio increased, while the LC3B II/I ratio decreased, indicating that the autophagy flux was blocked. Compared with the control and hypoxia groups, more LC3B type I was converted to type II during reoxygenation, and the P62 supernatant was restored with reduced sediment, confirming that autophagy was enhanced during reoxygenation (Figure 2(b)). Ambra1 was markedly decreased during hypoxia, then restored during reoxygenation but was significantly reduced in both conditions compared with the control group (Figure 2(c)). This indicated that Ambra1 may play an important role in regulating autophagy during hypoxia and reoxygenation. In conclusion, autophagy was blocked during hypoxia, then recovered after reoxygenation. Ambra1 was reduced during hypoxia and reoxygenation but was recovered during reoxygenation compared with that during hypoxia.

### 3.3. Blocking Autophagy during Both Hypoxia and Reoxygenation Was Detrimental

**Figure 3:**
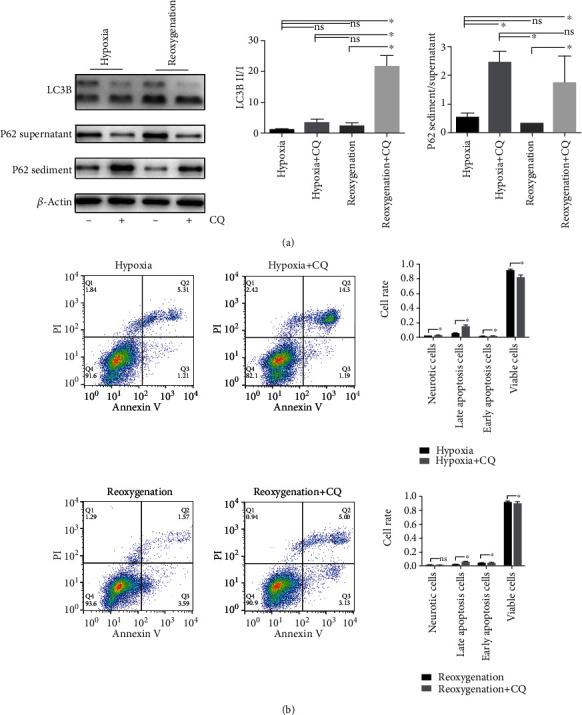
Blockage of autophagy flux by CQ causes worse outcome in H9C2 cell hypoxia and reoxygenation model. (a) Representative Western blot and quantitative evaluation of the expression of P62 sediment/supernatant and LC3B II/I. (b) Representative of flow cytometry image and quantitative evaluation of early apoptosis, late apoptosis, and neurosis. ^∗^*P* < 0.05; ns: no statistic difference.

To investigate the role of autophagy during hypoxia and reoxygenation, the autophagy inhibitor, chloroquine (CQ), was used to block autophagy by blocking autophagosome and lysosome fusion. Western blot results showed that when CQ was added, the P62 sediment increased as the supernatant decreased during hypoxia and reoxygenation compared with the no-CQ group, indicating that CQ successfully blocked autophagy flux (Figure 3(a)). We then used Annexin V and PI assays to evaluate necrosis and apoptosis in the hypoxia, hypoxia+CQ, reoxygenation, and reoxygenation+CQ groups via flow cytometry. Annexin V is a protein that can bind with phosphatidylserine (PS). Under normal conditions, PS is located on the inside of the cellular plasma membrane. During the early stages of apoptosis, PS translocates to the outer membrane and can combine with Annexin V. PI is a nucleic acid dye that cannot penetrate cells with intact plasma membranes such as normal cells and early-stage apoptotic cells. Therefore, normal cells cannot combine with Annexin V and PS. Cells can combine with Annexin but not PI in the early stages of apoptosis, but during the late stage of apoptosis, Annexin V and PI can be combined. Cells that can combine only with PI are necrotic. We found that blocking autophagy was harmful during both hypoxia and reoxygenation, as late apoptotic cells were significantly increased in the CQ groups, while viable cells were significantly decreased (Figure 3(b)). Thus, autophagy is crucial to protecting cells during H9C2 cell hypoxia and reoxygenation, and blocking autophagy can worsen the outcomes.

### 3.4. Ambra1 Reduced H9C2 Cell Damage during Both Hypoxia and Reoxygenation by Increasing Autophagy and Reducing ROS

**Figure 4:**
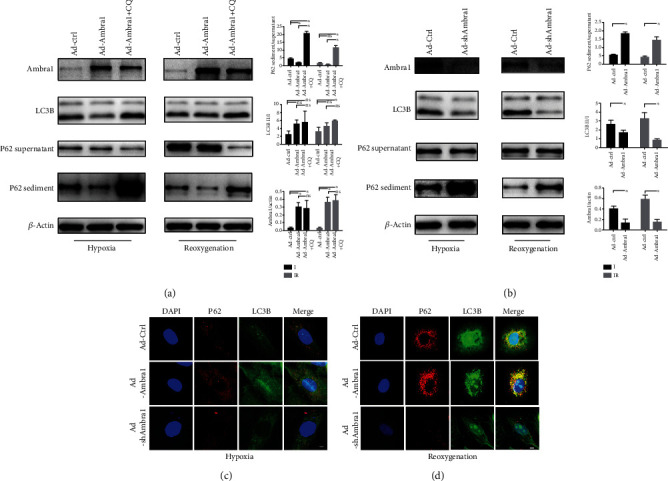
Ambra1 overexpression promotes autophagy while knockdown of Ambra1 blocks autophagy in H9C2 cell hypoxia and reoxygenation model. (a, b) Representative Western blot and quantitative evaluation of the expression of P62 sediment/supernatant, LC3B II/I, and Ambra1/actin. (c, d) Representative confocal image of P62 and LC3B in H9C2 cell hypoxia and reoxygenation model. Scale bar: 5 *μ*m; ^∗^*P* < 0.05; ns: no statistic difference.

**Figure 5:**
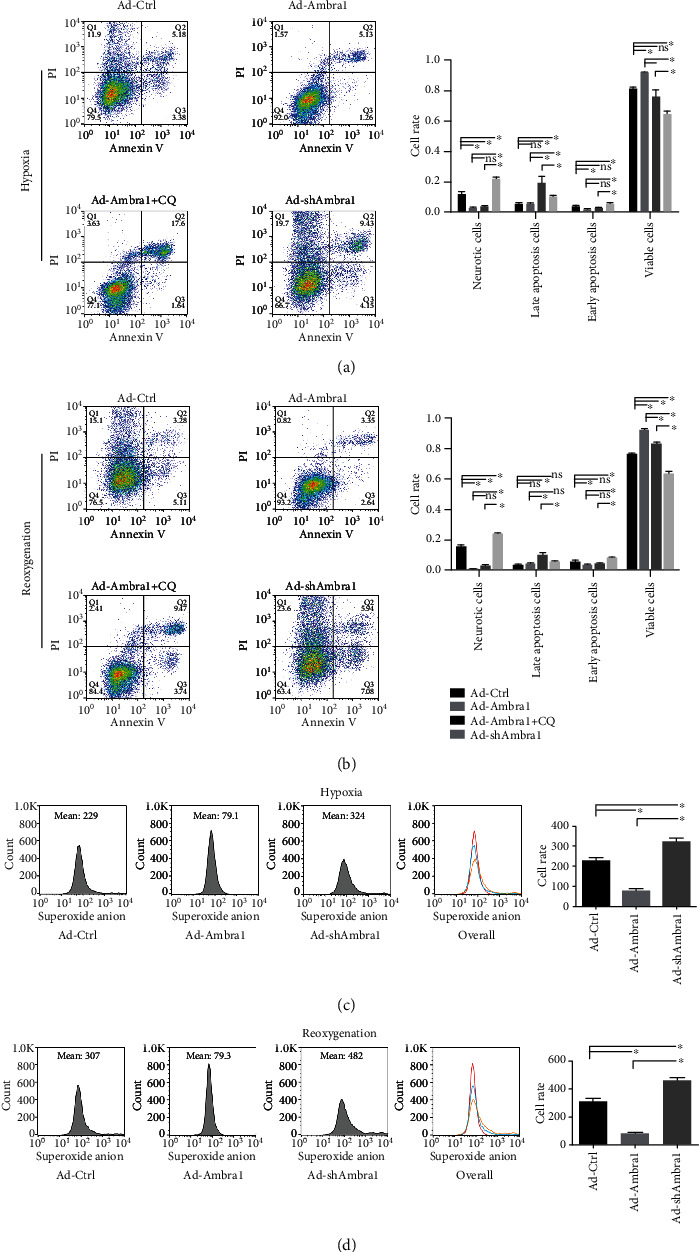
Ambra1 reduces H9C2 cell damage during both hypoxia and reoxygenation by increasing autophagy and reducing ROS. (a, b) Representative of flow cytometry image and quantitative evaluation of early apoptosis, late apoptosis, and neurosis. (c, d) Representative of ROS histogram and quantitative evaluation. ^∗^*P* < 0.05; ns: no statistic difference.

Because blocking autophagy resulted in poor outcomes for H9C2 cells during both hypoxia and reoxygenation and Ambra1 increased autophagy in H9C2 cells under basal condition, we decided to examine the role that Ambra1 plays in hypoxia and reoxygenation in H9C2 cells. We overexpressed Ambra1 by transfecting an Ambra1-overexpression adenovirus and used CQ to block autophagy flux by stopping the autophagosomes combining with lysosomes. We also knocked down Ambra1 to determine what occurs in H9C2 cells during hypoxia and reoxygenation. So we set up several groups: Ad-Ctrl group, Ad-Ambra1 group, Ad-Ambra1+CQ group, and Ad-shAmbra1 group. Western blotting showed that Ambra1 overexpression contributed to enhancing autophagy in both conditions, while CQ blocked autophagy flux successfully (Figure 4(a)). Conversely, knocking down Ambra1 blocked autophagy flux (Figure 4(b)). Confocal microscopy showed that increasing Ambra1 promoted LC3 combined with P62 in both conditions, and decreasing Ambra1 reduced this combination (Figures 4(c) and 4(d)). We detected H9C2 cell death, including necrosis and early-stage and late-stage apoptosis via flow cytometry, and Annexin V and PI dye were used to distinguish these situations. Consequently, during both hypoxia and reoxygenation, Ambra1 overexpression reduced necrosis and early apoptosis. Using CQ to block enhanced autophagy significantly increased late-stage apoptosis in both conditions. Knocking down Ambra1 significantly increased necrosis and apoptosis (Figures 5(a) and 5(b)). Thus, Ambra1 can reduce cell necrosis and apoptosis in H9C2 cell hypoxia and reoxygenation model via autophagy.

The exact causes of MIRI remain unclear, but extra ROS generation is an important reason that causes cell injury. As Ambra1 overexpression yielded better outcomes in H9C2 cell hypoxia and reperfusion model, and Ambra1 has been reported that it can reduce ROS through mitophagy, whether Ambra1 affects ROS in H9C2 cell hypoxia and reoxygenation model should be further explored. We used DHE to detect the superoxide anion. After living cells uptake DHE, it can be dehydrogenated by superoxide anions to produce ethidium. Ethidium produces red fluorescence when combined with RNA or DNA. The red fluorescence intensity parallels the ethidium level; thus, when the superoxide anion levels in cells are higher, more ethidium is produced. After transfecting the control, Ambra1-knockdown, and Ambra1-overexpression adenoviruses for 72 h, cells were incubated with DHE for 20 min in the dark; then, the red fluorescence was detected via flow cytometry and the mean fluorescence intensity was calculated. We found that overexpression of Ambra1 alleviated the superoxide anion regardless of hypoxia or reoxygenation, while decreasing Ambra1 aggravated superoxide anion production (Figures 5(c) and 5(d)). This indicated that Ambra1 can reduce superoxide anion in H9C2 cells during hypoxia and reoxygenation, thus protecting cells from oxidative damage.

### 3.5. Ambra1 Can Promote Autophagy by Improving the Stability and Activity of the ULK1 Complex

**Figure 6:**
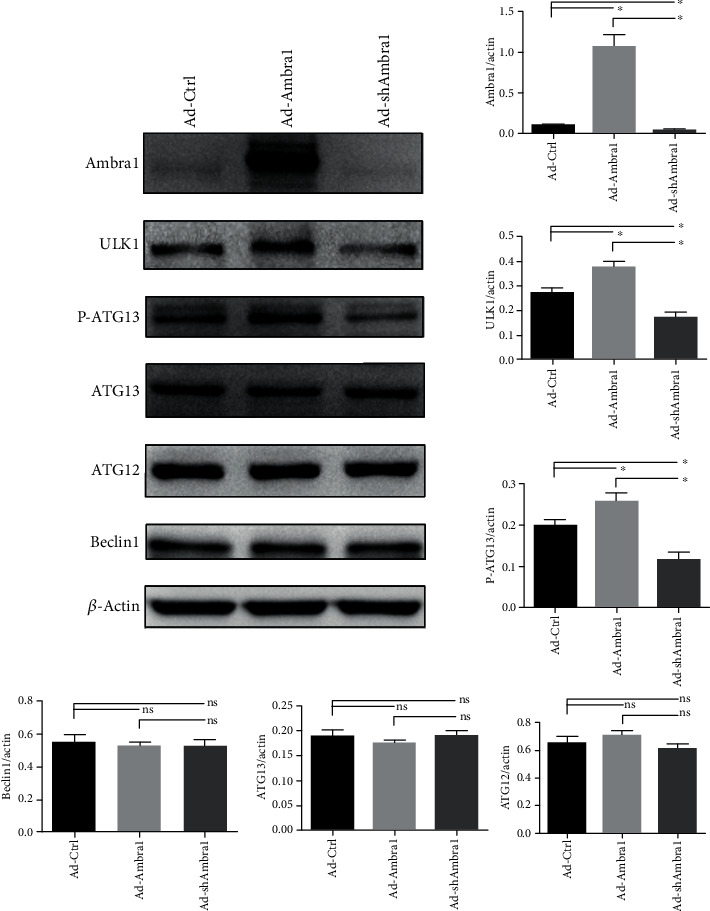
Ambra1 enhances autophagy in H9C2 cells by improving the stability and activity of the ULK1 complex. Representative Western blot and quantitative evaluation of the expression of Ambra1/actin, ULK1/actin, P-ATG13/actin, ATG13/actin, ATG12/actin, and Beclin1/actin. ^∗^*P* < 0.05; ns: no statistic difference.

In order to explore the mechanism by which Ambra1 promotes autophagy in H9C2 cells, we overexpressed Ambra1 by transfecting an Ambra1-overexpression adenovirus or reduced Ambra1 by transfecting an Ambra1-knockdown adenoviruses. As shown in Figure 6, we found that ULK1 increased when Ambra1 overexpressed and decreased when Ambra1 was knockdown, which shows that Ambra1 can enhance the stability of ULK1 complex. P-ATG13 changed similar to ULK1, as ULK1 phosphorylates ATG13 to promote autophagy initiation; this indicates that Ambra1 enhances ULK1 complex activity. Meanwhile, ATG12 and Beclin1 were not affected by the change of Ambra1 expression. This indicated that Ambra1 can improve the stability and activity of the ULK1 complex in H9C2 cells, and this effect may be specific, and this result has also been demonstrated in another cells by Nazio et al. [23].

## 4. Discussion

Here, we demonstrated for the first time the function of Ambra1 during hypoxia and reoxygenation in H9C2 cells, which mimics MIRI. Our results indicated that overexpressing Ambra1 enhanced autophagy in H9C2 cells in both physiological and pathological conditions, which benefits cell survival during both hypoxia and reoxygenation, and this outcome can be reversed by an autophagy inhibitor. Correspondingly, Ambra1 knockdown blocked autophagy flux and worsened the results in H9C2 cell hypoxia and reoxygenation. Moreover, Ambra1 reduced the superoxide anion during hypoxia and reoxygenation. Thus, Ambra1 may play several important roles in alleviating MIRI.

Ambra1 is a novel BH3-like protein that has been studied over the last decade [20] and plays a positive regulatory role in autophagy [33]. One part of Ambra1 is located on the dynein motor complex with Beclin1 and PI3KIII. Under normal cellular conditions, mTOR binds with ULK1 and phosphorylates it, further blocking autophagy. Under adverse conditions, such as starvation, mTOR dissociates from ULK1, then ULK mediates phosphorylation of the Ambra1, Beclin1, and PI3KIII complexes, which translocate to the endoplasmic reticulum, where the autophagosomes form. On the other hand, Ambra1 is a positive upstream regulator of ULK1 [34]. Under physiological conditions, mTOR also binds with Ambra1 at Ser 52 to phosphorylate it, further inhibiting its function in activating and stabilizing ULK1. Thus, a positive feedback loop may occur in ULK1-dependent regulation of Ambra1, and Ambra1 directly regulates ULK1 and Beclin1, playing a crucial role in favoring core complex formation in autophagy [23]. Our results indicated that under normal conditions, Ambra1 overexpression enhances autophagy flux, whereas Ambra1 deficiency blocks autophagy flux in H9C2 cells.

Autophagy in myocardial ischemia and reperfusion remains controversial, both in function and change. Our research showed that autophagy flux was blocked during hypoxia, then restored during reoxygenation, as the P62 sediment/supernatant ratio was increased with LC3B II/I ratio decreased during hypoxia. After reoxygenation, the LC3B II/I ratio was increased without increasing the P62 sediment/supernatant ratio, indicating that autophagy flux was restored. Our study showed that blocking autophagy by autophagy inhibitor, CQ, aggravated cell damage in both hypoxia and reoxygenation. Marek-Iannucci et al. also showed that increased autophagy plays a protective role in MIRI [31]. Conversely, Gao et al. revealed that increased autophagy worsened MIRI and caused more myocyte apoptosis and cardiac disfunction [30]. Matsui et al. showed that autophagy protects against hypoxia but is detrimental during reperfusion [35]. While Liu et al. showed that autophagy develops dynamically [36]. The differences between these studies may have been due to different experimental protocols, such as the ischemia and reperfusion duration, the facilities used to induce ischemia, and other influencing factors that remain unclear. Because Ambra1 promoted autophagy in H9C2 cells, we further detected the function of Ambra1 in an H9C2 cell hypoxia and reoxygenation model. Our results showed that Ambra1 contributed to increasing autophagy during ischemia and reoxygenation in H9C2 cells. After overexpressing Ambra1, the combination of P62 and LC3B increased. Cargo protein P62 combined with ubiquitinated proteins and interacted with LC3B on the autophagosomal membrane to form an autophagosome, then translocated to the perinucleus to be digested by lysosomes. This protective function can be overturned by the autophagy inhibitor, CQ, suggesting that autophagy is one reason that Ambra1 can mitigate H9C2 cell apoptosis and necrosis during hypoxia and reoxygenation. We further tested that knocking down Ambra1 reduced the P62 and LC3B combination and that autophagy was blocked and worsened the outcomes. The results supported that Ambra1 promoted autophagy, which is crucial to protecting myocytes in MIRI.

As ULK1 complex plays an important role in activating autophagy, and we demonstrated that in H9C2 cells, an increase in Ambra1 results in an increase in ULK1. Further, P-ATG13 was also increased in this situation while there are no difference in ATG12 and Beclin1. All of this indicates that Ambra1 improves the stability and activity of the ULK1 complex, and this effect is exclusive. Bartolomeo et al. has shown that ULK1 kinase can phosphorylate Ambra1 thus release the autophagy core complex from dynein [22, 37]. Therefore, a positive feedback loop may be formed between ULK1 and Ambra1, and Ambra1 enhances autophagy in H9C2 cells by improving the stability and activity of the ULK1 complex.

Myocardial ischemia and reperfusion injury are severe problems in blood restoration and may counteract the benefit of reperfusion therapy [5]. The exact reasons for MIRI remain unclear, but several issues are thought to cause MIRI [15], of which, oxidative stress is considered one of the most important [38]. ROS are produced in cardiomyocytes during ischemia due to the residual oxygen. In the early stage of reperfusion therapy with blood supply restoration, the oxygen supply is also restored, causing a burst of ROS. Excessive ROS results from imbalanced free radical production and elimination, thus damaging DNA, proteins, and lipids and further causing cell necrosis and apoptosis. We found that overexpressing Ambra1 reduced ROS during hypoxia and reoxygenation, while knocking down Ambra1 exacerbated ROS. Di Rita et al. reported a similar observation in SH-SY5Y cells: Ambra1 significantly reduced ROS production induced by 6-OHDA and rotenone [25]. This indicates that Ambra1 may be a potential target for treating MIRI. Strappazzon et al. reported that Ambra1 can contribute to mitophagy by directly binding with LC3, regardless of whether P62 and PARKIN participate [24]. Mitophagy is a specific autophagy that removes damaged mitochondria, as damaged mitochondria are an important resource for ROS in MIRI. We suspect that Ambra1 can reduce ROS through mitophagy to further alleviate MIRI.

Our research explored the function of Ambra1 in MIRI on H9C2 cells. Overexpression and knockout of Ambra1 in animal models are closer to the MIRI pathophysiology. Autophagy flux, ROS, cell necrosis, and apoptosis detected in vivo will better reveal the role that Ambra1 plays in MIRI. Our further research will focus on these issues.

## Data Availability

The data used to support the findings of this study are available from the corresponding author upon request.
